# Resurgence of Clinical Malaria in Ethiopia in the Era of Anopheles stephensi Invasion

**DOI:** 10.21203/rs.3.rs-4468361/v1

**Published:** 2024-06-12

**Authors:** Guofa Zhou, Hiwot S. Taffese, Daibin Zhong, Xiaoming Wang, Ming-Chieh Lee, Teshome Degefa, Dejene Getachew, Werissaw Haileselassie, Dawit Hawaria, Delenasaw Yewhalaw, Guiyun Yan

**Affiliations:** University of California Irvine; Ethiopia Ministry of Health; University of California Irvine; University of California Irvine; University of California Irvine; Jimma University; Adama Science and Technology University; Addis Ababa University; Hawassa University; Jimma University; University of California Irvine

**Keywords:** Clinical malaria, An. stephensi, urban area, outbreak, hot spot, knowledge gap

## Abstract

**Background.:**

The invasion of *Anopheles stephensi* into Africa poses a potential threat to malaria control and elimination on the continent. However, it is not clear if the recent malaria resurgence in Ethiopia has linked to the expansion of *An. stephensi*. We aimed to summarize the major achievements and lesson learnt in malaria control in Ethiopia from 2001 to 2022, to assess the new challenges and prospects for the control of *An. stephensi*.

**Methods and findings.:**

We obtained the clinical malaria case reports, antimalarial drug treatment records, insecticide-treated and long-lasting insecticidal net (ITN/LLIN) distribution and utilization records, and indoor residual spraying (IRS) coverage data from the Ethiopian Ministry of Health (MoH) for the period 2001–2022. We analyzed clinical malaria hotspots using spatially optimized hotspot analysis. We investigated malaria outbreaks in 2022 and examined the potential role of *An. stephensi* in the outbreaks.

Clinical malaria cases in Ethiopia decreased by 80%, from 5.2 million cases (11% confirmed) in 2004 to 1.0 million cases (92% confirmed) in 2018; however, cases increased steadily to 2.6 million confirmed cases (98% confirmed) in 2022. *Plasmodium vivax* cases and proportion have increased significantly in the past 5 years. Clinical malaria hotspots are concentrated along the western Ethiopian border areas and have grown significantly from 2017 to 2022. Major malaria outbreaks in 2022/23 were detected in multiple sites across Ethiopia, and *An. stephensi* was the predominant vector in some of these sites, however, it was absence from many of the outbreak sites.

**Conclusions.:**

The malaria burden has been significantly reduced in Ethiopia in the past two decades, but in recent years it has increased substantially, and the cause of such increase is a subject of further investigation. Major gaps exist in *An. stephensi* research, including vector ecology, surveillance, and control tools, especially for adult mosquito control.

## Background

Malaria morbidity and mortality have decreased significantly in the past two decades due to the scale-up of interventions, but malaria remains the most serious tropical infectious disease globally [1]. Worldwide in 2022, there were an estimated 249 million malaria cases in 85 malaria endemic countries and areas, an increase of 5 million cases compared with 2021, deaths declined in 2022 to 608,000 from 631,000 in 2020 [1]. In Ethiopia, malaria transmission has been a significant cause of public health issues due to its pronounced seasonal and regional fluctuation. Ethiopia is one of the few countries in Africa where *Plasmodium falciparum* and *Plasmodium vivax* are both endemic [2], making the malaria control and elimination more complex than the other malaria endemic African countries. Reported malaria cases decreased from a peak of 5.2 million in 2004 to 1.0 million in 2018 [1, 2]. Encouraged by the progress made since 2000, Ethiopia envisaged the elimination of malaria and began a subnational program in 2017 and expanded to the national level in 2021, setting the goal of zero indigenous malaria cases by 2030 [3]. Despite these major achievements, there has been an upsurge in malaria burden in Ethiopia in the past few years. The Ethiopian Ministry of Health (MoH) has confirmed 2.6 million malaria cases in 2022. In addition, the invasion of *Anopheles stephensi* Liston 1901 (Diptera : Culicidae), a malaria vector from South Asia, into Africa – with Ethiopia as the epicenter – and its rapid expansion in its new territory pose a significant threat to malaria control and elimination on the continent [4–8]. Since it was reported from Djibouti in 2012, *An. stephensi* has been detected in Ethiopia, Eritrea, Sudan, Somalia, Kenya, Nigeria, and Ghana [9]. The invasion of An. stephensi has been linked to malaria outbreaks in the urban areas of Djibouti and Ethiopia [10–13]. The World Health Organization (WHO) has called for urgent action to halt the spread of *An. stephensi* in Africa [9].

The Ethiopian MoH has updated its vector control policy in response to the new threat. To contain the spread of *An. stephensi*, the Ethiopian MoH partnered with The President’s Malaria Initiative (PMI) launched a larval source management (LSM) program using microbial larvicide for *An. stephensi* control in eight cities in northeastern and central Ethiopia [14]. However, major knowledge gaps on *An. stephensi* ecology, behavior and its containment exist which may hamper vector control planning.

The aim of this study was to summarize the major achievements, challenges, and knowledge gaps in malaria control in Ethiopia from 2001 to 2022, to assess the changes in malaria epidemiology from 2017 to 2022, to document the new challenges in malaria control due to the invasion of *An. stephensi*, and to discuss the prospects for its control. The findings will be useful for guiding policy updates to contain the spread of *An. stephensi* and to minimize its impact on malaria transmission in Africa and specifically in Ethiopia, the current epicenter of the invasion.

## Materials and Methods

We obtained the clinical malaria case reports, microscopy examination and rapid diagnostic test (RDT) results, antimalarial drug treatment records, insecticide-treated and long-lasting insecticidal net (ITN/LLIN) distribution records, and indoor residual spraying (IRS) coverage data from the Ethiopian MoH for the period 2001–2022. The nationwide clinical case reports included weekly microscopy and RDT confirmed cases, probable cases (clinically diagnosed), and parasite species at each woreda (administrative unit equivalent to district or county). Microscopy and RDT results included the number of tested cases, positive cases, and parasite species. Malaria treatment drugs delivered included artemisinin combination therapies (ACTs) and others. ITN/LLIN records included the number of nets distributed, and IRS data included population coverage.

**Figure 1:**
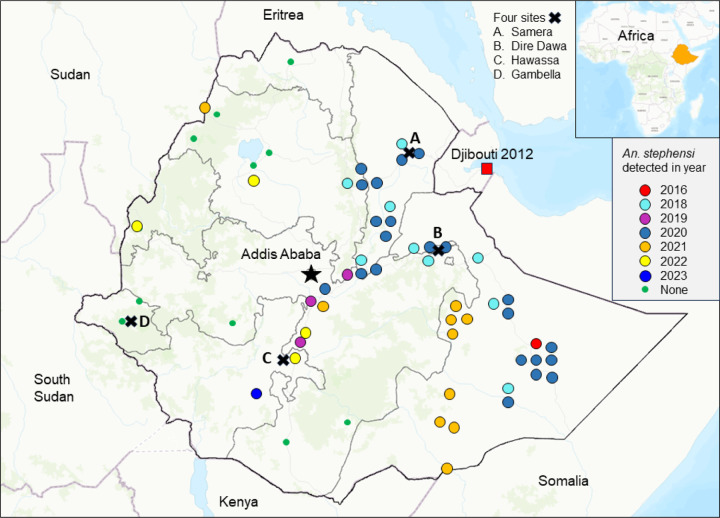
Distribution map of *An. stephensi* from 2016 to 2023 and the locations of the four study sites (shown in Figure 4) in Ethiopia.

We obtained the updated *An. stephensi* records, including *An. stephensi* distribution and year of detection, from the MoH, PMI, our field work (10 sites), and other published work (Fig. 1) [4–6, 15–18]. The proportion of *An. stephensi* included adults from reared larvae and from adult surveys.

To assess disease hotspots and their changes from 2017 to 2022, we analyzed woreda-level malaria incidence rates (cases/1,000 persons at risk/year) based on the weekly outpatient records and population projections from the Ethiopia office of the United Nations Office for the Coordination of Humanitarian Affairs (https://data.humdata.org/organization/ocha-ethiopia). We selected the period 2017–2022 for two reasons. Firstly, the changes in malaria incidence distribution and malaria management policy in Ethiopia from 2013 to 2016 have been studied [2]. Secondly and more importantly, *An. stephensi* was first detected in Kebri Dehar in the Somali Regional State in eastern Ethiopia in 2016, and 2017 could be seen as a key turning point for urban malaria in Ethiopia, as *An. stephensi* was detected in 9 other sites across eastern Ethiopia in 2018 (Fig. 1) [5]. We determined hotspots and coldspots of clinical cases using the Optimized Hot Spot Analysis tool of ArcGIS 10.8.2 (ESRI, Redlands, CA 92373, USA), which calculates Getis-Ord Gi* spatial statistics and z-scores at significance levels of 90%, 95%, and 99% [19, 20]. We analyzed malaria risk level changes from 2017 to 2022 based on the Ethiopian MoH risk classifications using annual parasite incidence (API), i.e., malaria free API = 0, very low risk 0 ~ 5, low risk 5 ~ 10, moderate risk 10 ~ 50, and high risk ≥ 50 annual parasite infection rate per 1,000 people.

To analyze clinical malaria outbreaks, we randomly selected 40 districts representing different regions of Ethiopia, with at least three sites for each administrative region. We obtained the monthly confirmed malaria cases for the 40 sites for the period 2013–2022. We used Cullen’s method to detect malaria outbreak months from 2018 to 2022 based on 2013–2017 case numbers adjusted for population growth [21]. After initial evaluation of data completeness, 33 sites were included in the data analysis. The analysis of variance (ANOVA) with repeated measure was used to compare monthly clinical malaria incidences between 2022 and 2017–2021. We used the proportion of *An. stephensi* at each site to examine if *An. stephensi* played any role in malaria outbreaks in 2022.

## Results

### Malaria epidemiology, diagnosis, treatment, and prevention in Ethiopia

**Figure 2:**
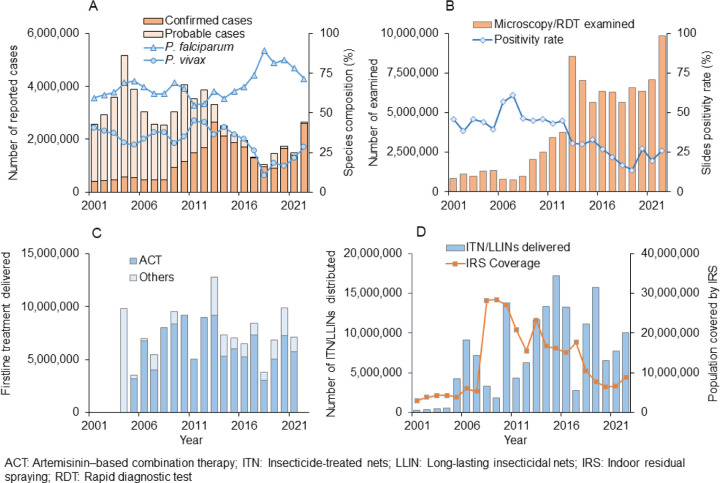
A: Reported clinical malaria cases and parasite species composition from 2001 to 2022; B: Number of RDT and microscopically examined blood samples and slide positivity rate from 2001 to 2022; C: Antimalarial drugs delivered from 2005 to 2022; and D: ITN/LLIN distribution and IRS population coverage from 2001 to 2022.

Nationwide, reported clinical malaria cases (probable plus confirmed) remained unchanged from 2001 to 2013, although with great fluctuations, and most of the reported cases were probable cases (Fig. 2A). This was followed by a significant decline from 2013 (2.65 million confirmed cases and 0.67 million clinical cases) to 2018 (0.96 million confirmed cases and 0.08 million clinical cases). However, clinical malaria cases have increased slowly but steadily since 2018, reaching 2.65 million cases (2.60 million confirmed cases and 0.05 million clinical cases) in 2022 (Fig. 1A). The proportion of *P. falciparum* malaria cases was stable from 2001 to 2014 and increased from 2014 to 2018, while the proportion of *P. vivax* cases increased from 10.6% in 2018 to 28.4% in 2022 (Fig. 2A), reflecting a 7-fold increase in vivax malaria cases from 2018 (0.10 million) to 2022 (0.73 million). The increase in clinical malaria cases was clearly not due to the lack of diagnostic tools, because the number of microscopically and RDT examined blood samples was stable from 2013 to 2021 (average of 6.5 million per year) and increased significantly to 9.8 million in 2022 (Fig. 2B). Distribution of antimalarial drugs fluctuated but remained relatively stable from 2006 to 2022 (Fig. 2C). The number of ITNs/LLINs delivered fluctuated greatly, with the major mass distributions occurring in 2007, 2010, 2015, and 2019 (Fig. 2D). Per Ethiopian government policy, IRS was implemented in epidemic-prone areas of the country. Therefore, the reduced IRS coverage from 2009 (28.37 million people) to 2022 (8.86 million people) is likely a reflection of reduced malaria risk levels rather than reduced effort (Fig. 2D).

### Changes in clinical malaria incidence from 2017 to 2022

**Figure 3:**
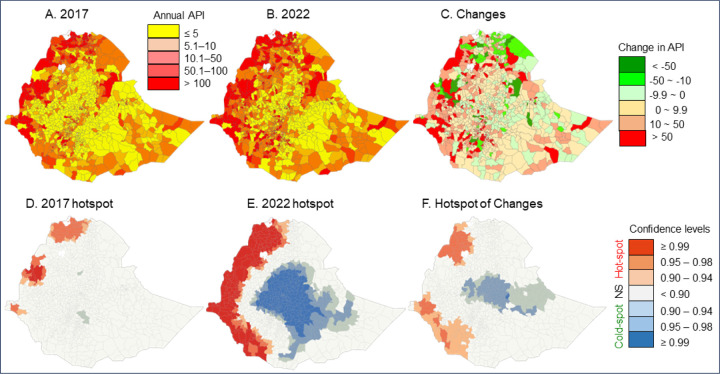
Top panel: Map of malaria annual parasite incidence (API) distribution in 2017 (A), 2022 (B), and changes from 2017 to 2022 (C). Bottom panel: The corresponding hot/cold spots detected from the API distribution.

Malaria risk levels increased significantly in many districts from 2017 to 2022, based on the changes in API (Table 1, Figs. 3A, B). For example, in 2017 there were 47 districts and 7.19 million people free of malaria (API = 0); by 2022, malaria-free areas had decreased to 19 districts and 2.42 million people (Table 1). The number of districts with high malaria risk increased from 161 districts (8.33 million people) in 2017 to 267 woredas (15.88 million people) in 2022 (Table 1). It is important to note that not all districts had an increased risk. For example, 30 districts where malaria risks were high in 2017 had decreased risk levels in 2022, while 150 districts with lower risk levels in 2017 jumped to high-risk levels in 2022 (Table 1, Figs. 3A–C). Overall, from 2017 to 2022, malaria incidence rates decreased in 38 districts covering a population of 22,965,183 people, while malaria incidence rates increased in 780 woredas covering a population of 76,626,859 people. Malaria incidence remained unchanged (defined as change in malaria incidence rate < 0.1 cases/1,000 people/year) in 38 woredas covering a population of 5,757,660 people.

Clinical malaria hotspots in 2017 were concentrated in three focal areas along the western Ethiopia borders, and no clear coldspots were detected (Fig. 3D). By 2022, hotspot areas had expanded tremendously along the western borders and a large coldspot was detected in central Ethiopia (Fig. 3E), revealing an increase in clinical malaria incidence in western Ethiopia. Hotspots of increase in clinical malaria occurred in northwestern and southwestern Ethiopia (Fig. 3F).

#### Malaria outbreaks and the role of An. stephensi

**Figure 4:**
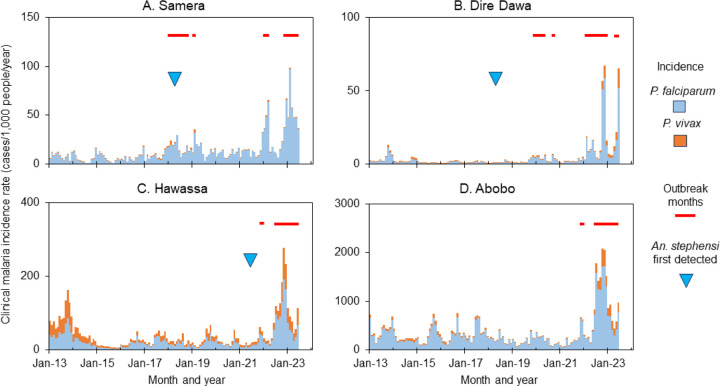
Example of dynamics and outbreaks detection in clinical malaria in selected study sites from 2013 to 2022.

**Figure 5:**
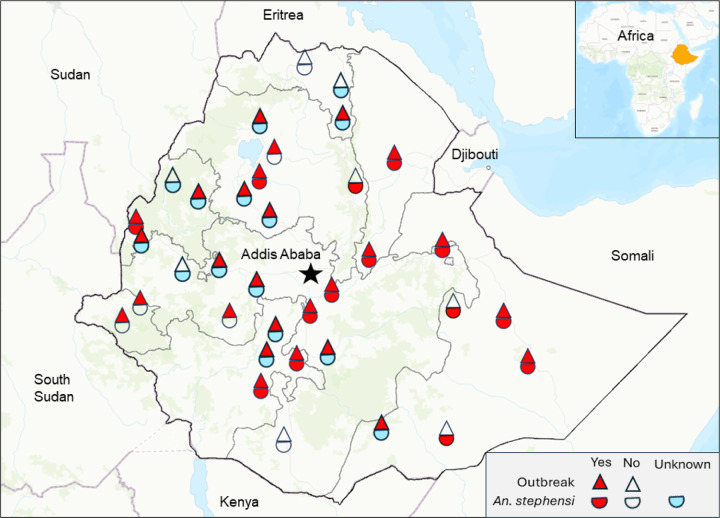
Results of outbreak detection at the 33 selected sites and *An. Stephensi* presence status.

Ten-year dynamics of clinical malaria showed a strong heterogeneity (Figs. 4 & S1). Trend of clinical malaria dynamics can be classified into four categories (Figure S1), i.e., sudden recent outbreaks such as in Dire Dawa, declined trend with recent resurgence such as in Adama, stable trend with seasonality such as in Alamata, and overall declining trends such as in Abeshega and Yebelo (Figure S1). Malaria incidence rate increased significantly from 2017 to 2022 in many study sites, while it decreased significantly in other sites (supplement Table S1). For example, clinical malaria incidence rates in Dire Dawa increased 9.0-fold in 2022 compared to the average of 2017–2022 (ANOVA with repeated measure, P < 0.01), while in Kemise Town it decreased about 40% during the same period (P < 0.01, Table 2). Overall, malaria outbreaks were detected in 25 of the 33 selected sites (Figs. 4, 5 & S1, Table 2), indicating the severity of malaria outbreaks in Ethiopia in 2022.

The link between *An. stephensi* and malaria outbreaks was complex (Table 2). The proportion of *An. stephensi* in some outbreak sites was high, for example, in Semera and Dire Dawa, *An. stephensi* accounted for 96–100% of all *Anopheles adults* collected in 2022, indicating a possible contribution of *An. stephensi* to the outbreaks (Table 2). Whereas, in Fik, no other *Anopheles* have been collected in 2022 except *An. stephensi,* there was a significant decrease in clinical malaria incidence in 2022 compared to 2017–2021 (Table 2). Furthermore, in many places where malaria outbreaks have been detected in 2022, for example, in Abobo and Gambella, no *An. stephensi* has been detected so far (Table 2), i.e., outbreaks in some sites were independent of the existence of *An. stephensi*.

## Discussion

Malaria morbidity and mortality have significantly decreased from over 5 million clinical cases in early 2000 to about 1 million in 2018. Encouraged by the significant progress that has been achieved, the Ethiopian National Malaria Eradication Program (NMEP) has set the goal of achieving zero indigenous malaria in the country by 2030. However, the COVID-19 pandemic may have paused the declining trend in clinical malaria. The increase in clinical malaria since 2019 and the sudden malaria outbreak in Ethiopia in 2022–23 are alarming signs for malaria control. Still more ominous is the emergence and rapid spread of the highly efficient and invasive vector *An. stephensi*, which may jeopardize the gains made in malaria control in the past decade. While native African malaria vectors breed mainly in rural natural habitats, *An. stephensi* demonstrates a robust ability to breed and thrive in urban environments, leading to malaria outbreaks in urban areas [12, 13, 22]. Clearly, *An. stephensi* is not the cause of the 2022 malaria outbreak in Ethiopia because malaria outbreak occurred in many places where *An. stephensi* has not been detected, however, malaria outbreaks in some urban areas might be associated with *An. stephensi*, because *An. stephensi* was nearly the sole malaria vector in some areas. The recent upsurge in clinical malaria in Ethiopia may be multi-factorial including climate change, deterioration of healthcare system due to the COVID-19 pandemic, civil unrest in northwestern Ethiopia, and refugees in southwestern Ethiopia among other factors [23–31]. Other possible causes for the 2022 malaria outbreaks include but are not limited to the possible K13 gene mutation related antimalarial drug resistance by malaria parasite and potential missed diagnosis of *P. falciparum* infections due to the PfHRP2/3 deletions, both need further investigations. Nonetheless, although the contribution of *An. stephensi* to malaria outbreaks in 2022 is a subject for further investigation [7, 11], the outbreaks indicate the severity of the situation.

The WHO has reported a global increase in malaria cases in 2022; and Pakistan, Ethiopia and Nigeria were the three countries with increase of > 1 million malaria cases from 2021 to 2022 [32]. The causes of the recent upsurge in clinical malaria in Ethiopia are worth of in-depth investigations. Although its contribution is a subject of further investigation in Ethiopia, climate change might have major impact on malaria transmission and risk globally [33–35]. Civil unrest in northwestern Ethiopia caused the setting up of many internally displaced people’s camps and the influx of Sudanese refugees in southwestern Ethiopia (mainly in Gambella Region) led to the setup of refugee camps in the area, *Plasmodium* infections are prevalent in these camps which may serve as reservoirs for local transmission [30, 31, 36]. More importantly, COVID-19 could have contributed to the malaria resurgence and outbreak by an accumulative effect [37–39]. COVID-19 pandemic interrupted not only the services at health facilities but also peoples’ malaria treatment seeking behavior. Without effective treatment *Plasmodium* parasite reservoir might have cumulated from 2020 to 2021 and eventually caused the malaria outbreaks in Ethiopia in 2022, this hypothesis needs to be investigated. Although Ethiopian MoH has implemented the primaquine 14-day low-dose radical treatment of *P. vivax* since 2021, there was an increased proportion of clinical vivax malaria cases in 2022, the causes of such an increase requires further investigation. Lastly, we cannot rule out the contribution of *An. stephensi* in malaria outbreaks in Ethiopia because *An. stephensi* was the predominant in come urban areas in Ethiopia [11, 12, 15].

To contain the spread of *An. stephensi* in Africa, WHO has announced an initiative to support an effective response to *An. stephensi* on the African continent in 2022 [9]. The WHO initiative sets five aims, including increasing collaboration among national malaria control programs, researchers, and funders to ensure sharing of knowledge, optimization of resources, and prioritization of key activities. The Ethiopian MoH, in partnership with PMI, has worked with the NMEP and researchers on enhanced surveillance to document the spread and vectorial capacity for malaria transmission in Ethiopia. They have launched LSM programs in eight cities in Ethiopia aiming to reduce *An. stephensi* populations and to slow and eventually prevent its spread. However, there are major knowledge gaps and policy implications to consider.

The WHO recommends strengthening surveillance, including entomological surveillance, which can determine the spread of *An. stephensi* and its role in transmission, and malaria case surveillance, which can be used to investigate the impact of *An. stephensi* on malaria, particularly in urban areas. However, the optimal sampling method for *An. stephensi* adult mosquitoes has not yet been established. For example, aspiration (suction applied by a human or machine) is often used for An. stephensi adult samplings [15], but this method is highly subjective regarding the selection of sampling locations, i.e., one may intentionally select potential *An. stephensi* resting places such as animal shelters. Human landing catches (HLC) is considered the gold-standard for African *Anopheles* adult samplings [40–42], it will have similar problems; e.g., should one sit inside/outside human dwellings or inside/outside animal shelters? Similarly, for larval surveys one may deliberately select container habitats instead of randomly selecting both man-made and natural habitats. More importantly, these sampling methods may affect the population dynamics assessments [15, 16]. Additionally, we must take into consideration of native African malaria and arboviral disease vectors, as they are present in some urban areas and may require different sampling methods than *An. stephensi*. Since we found most *An. stephensi* adults inside animal shelters, animal-baited traps may be an efficient trapping method, but it must be thoroughly evaluated under semi-field and field conditions. In any case, the sampling method(s) with less bias and better efficiency for both native African malaria vectors and *An. stephensi* need to be carefully investigated.

The WHO also recommends prioritizing research; specifically, it recommends evaluating the impact of vector control interventions, particularly new tools, against An. stephensi and focuses on research which will enable malaria control programs to find better ways to respond to this invasive vector. The Ethiopian government and PMI are conducting pilot larviciding programs using *Bacillus thuringiensis* var. *israelensis* (*Bti*) in a number of cities, aiming to generate data for strategic control of *An. stephensi*. This is a good starting point, as *An. stephensi* breeds mostly in man-made habitats such as artificial water containers and construction pits, however it is hard for LSM to cover all larval breeding habitats. Therefore, adult control tools should also be developed and evaluated. Since we know that *An. stephensi* rests mainly in animal shelters or other outdoor structures in Ethiopia, we can develop new mosquito control methods utilizing this information [43]. For example, targeting animal shelters with IRS may significantly reduce the population density of *An. stephensi*. Regardless, integration of different control tools and including both larval and adult interventions may help to greatly reduce the *An. stephensi* population if not eliminate it.

There are limitations regarding the clinical malaria case reporting and the investigations of the causes of the 2022 malaria outbreaks in Ethiopia. In Ethiopia, private clinics also provide service for malaria diagnosis and treatment, however, compared to the free malaria treatment at government-run public healthcare facilities, the cost at private clinics may prevent some patients from seeking treatment over their facilities. Therefore, the under-report of clinical malaria cases from private clinics may not be a major issue. The causes of the 2022 malaria outbreaks in Ethiopia may be multi-factorial, which requires further investigations, and this can be a major study itself. Another limitation is the clinical malaria case dynamics at each woreda, it would be better to used incidence rate based on population size, however, because the Ethiopian government has not done any census since 2007 and some woredas have been redraw in the past 10 years, therefore it is very difficult to calculate incidence rate over time.

In conclusion, while the malaria burden in Ethiopia has been greatly reduced in the past 20 years, the 2022–23 malaria outbreak has undercut control efforts. Although the overall contribution of *An. stephensi* to malaria transmission in Africa is unclear, it has been linked to the malaria outbreaks in some urban settings of Africa. Larviciding has been implemented in Ethiopia for controlling *An. stephensi*, but adult control strategies should also be developed. The rapid growth of many African cities and global climate change, coupled with the invasion and spread of this highly efficient and adaptable malaria vector and the knowledge gaps surrounding it, could undermine the malaria control and elimination efforts in Ethiopia and other African countries. Containing the spread of *An. stephensi* and eliminating malaria in Africa requires strong international collaboration, investment, and commitment.

## Figures and Tables

**Table 1:** Changes in at-risk populations from 2017 to 2022

Risk level	At risk woredas and population				Risk changes from 2017–2022			
	2017		2022		Reduced †		Increased †	
	N Woreda	Population	N Woreda	Population	N Woreda	Population	N Woreda	Population
API = 0	47	7,189,228	19	2,422,574	0	0	28	4,766,654
API ≤5	477	58,183,932	339	43,763,037	130	15,089,712	328	41,383,701
5 < API < 10	148	13,093,116	127	13,165,220	33	2,294,447	106	11,187,439
10 ≤ API < 50	251	18,552,599	332	30,115,271	72	4,242,569	235	21,296,553
API ≥ 50	161	8,330,827	267	15,883,600	30	1,338,454	150	8,516,825
Total					265	22,965,183	780	76,626,859

† API values indicate the reduction/increase in API ranges. API: annual parasite incidence

**Table 2:** Malaria outbreak detection and *An. stephensi* status and proportion in selected sites

Region/Woreada	Outbreak detection			*Anopheles stephensi*	
	Status	Rate ratio	P-value	Status	Proportion
Afar					
Awash Fentale	No	0.87	0.0573	Yes	92%
Semera	Yes	1.83	0.0334	Yes	100%
Amhara					
Bahir Dar Town	Yes	2.69	0.0088	No	0%
Debre Markos Town	Yes	2.46	0.0069	No	0%
Gondar Zuriya	Yes	3.41	0.0007	No	0%
Kemise Town	No	0.60	0.0036	No	0%
Woreta	Yes	2.12	0.0297	No	0%
Benishangul-Gumuz					
Assosa	Yes	1.47	0.0116	No	0%
Bambasi	Yes	1.66	0.0013	No	0%
Guba	No	0.69	0.0689	NA	
Pawe	Yes	1.62	0.0783	NA	
Dire Dawa					
Dire Dawa	Yes	9.04	0.0093	Yes	96%
Gambella					
Abobo	Yes	4.02	0.0020	No	0%
Gambella	Yes	1.60	0.0112	No	0%
Oromia					
Adama	Yes	1.64	0.1161	Yes	NA
Batu	Yes	2.21	0.0328	Yes	77%
Yabelo	No	0.75	0.0651	NA	
Jimma Town	Yes	3.82	0.0030	No	0%
Nekemte Town	Yes	2.73	0.0021	NA	
Negele Town	Yes	6.04	0.0006	NA	
Gimbi	Yes	6.31	0.0029	NA	
Southern Nations, Nationalities and Peoples					
Arba Minch	Yes	1.18	0.1050	Yes	NA
Abeshige	Yes	1.32	0.1793	NA	
Shashago	Yes	1.36	0.0314	NA	
Hawassa Town	Yes	3.01	0.0303	Yes	62%
Somali					
Fik	No	0.69	0.0054	Yes	100%
Filtu	No	0.70	0.0013	NA	
Godey	No	0.88	0.1020	Yes	64%
Degehabur	Yes	1.01	0.4818	Yes	58%
Kebri Dehar	Yes	1.52	0.0063	Yes	75%
Tigray					
Mekele	NA	NA	NA	No	0%
Tahitay	NA	NA	NA	NA	
Alamata	Yes *	NA	NA	NA	

Rate ratio: Incidence rate of 2022 over average of 2017–2021

Proportion of *An. stephensi:* Combination of larval-reared adults and adult collections.

NA: Not available

* Outbreak in 2020

## Data Availability

The data underlying this publication are available freely upon request, complying with the data access policies of participated institutions.

## Abbreviations

ACT: artemisinin combination therapies
ANOVA: analysis of variance
API: annual parasite incidence
Bti: Bacillus thuringiensis var. israelensis
HLC: human landing catches
IRS: indoor residual spraying
ITN: insecticide-treated bed net
LLIN: long-lasting insecticidal net
LSM: larval source management
MoH: Ministry of Health
NMEP: National Malaria Eradication Program
PMI: the President’s Malaria Initiative
RDT: rapid diagnostic test
WHO: World Health Organization
